# Are perinatal measures associated with adolescent mental health? A retrospective exploration with original data from psychiatric cohorts

**DOI:** 10.1186/s12888-022-04302-6

**Published:** 2022-10-28

**Authors:** Lukas A. Basedow, Sören Kuitunen-Paul, Veit Roessner, Gunther H. Moll, Yulia Golub, Anna Eichler

**Affiliations:** 1 Faculty of Medicine, Department of Child and Adolescent Psychiatry, Technische Universität Dresden, Fetscherstrasse 74, 01307 Dresden, Germany; 2 Dept. of Psychology, Division of Clinical Psychology and Psychotherapy, Philipps-University of Marburg, Marburg, Germany; 3 Chair for Clinical Psychology and Psychotherapy, Technische Universität Chemnitz, Chemnitz, Germany; 4 Department of Child and Adolescent Mental Health, Faculty of Medicine, Friedrich-Alexander-Universität Erlangen-Nürnberg (FAU), Schwabachanlage 6, 91054 Erlangen, Germany

**Keywords:** Addiction, Adolescents, Mental Disorders, Apgar, Birth, Perinatal

## Abstract

**Background:**

Perinatal markers of prenatal development are associated with offspring psychiatric symptoms. However, there is little research investigating the specificity of perinatal markers for the development of specific disorders. This study aimed to explore if perinatal markers are specifically associated with adolescent substance use disorder (SUDs).

**Methods:**

Adolescent participants from two study centers, one for SUD patients (*n* = 196) and one for general psychopathology (*n* = 307), were recruited for participation. Since the SUD participants presented with a number of comorbid disorders, we performed a 1-on-1 matching procedure, based on age, gender, and specific pattern of comorbid disorders. This procedure resulted in *n* = 51 participants from each group. From all participants and their mothers we recorded perinatal markers (mode of birth, weeks of completed pregnancy, birth weight, Apgar score after 5 min) as well as intelligence quotient (IQ). The SUD sample additionally filled out the Youth Safe Report (YSR) as well as the PQ-16 and the DUDIT. We aimed to distinguish the two groups (SUD sample vs. general psychiatric sample) based on the perinatal variables via a logistic regression analysis. Additionally, linear regressions were performed for the total group and the subgroups to assess the relationship between perinatal variables and IQ, YSR, DUDIT and PQ-16.

**Results:**

The perinatal variables were not able to predict group membership (*X*^*2*^ [4] = 4.77, *p* = .312, Cox & Snell R² = 0.053). Odds ratios indicated a small increase in probability to belonging to the general psychiatric sample instead of the SUD sample if birth was completed via C-section. After Bonferroni-correction, the linear regression models showed no relation between perinatal markers and IQ (*p* = .60, R² = 0.068), YSR (*p* = .09, R² = 0.121), DUDIT (*p* = .65, R² = 0.020), and PQ-16 (*p* = .73, R² =0.021).

**Conclusion:**

Perinatal markers were not able to distinguish SUD patients from patients with diverse psychopathologies. This pattern contradicts previous findings, perhaps because our chosen markers reflect general processes instead of specific mechanistic explanations. Future studies should take care to investigate specific prenatal markers and associate them with psychopathology on the symptom level.

**Supplementary Information:**

The online version contains supplementary material available at 10.1186/s12888-022-04302-6.

## Background

The development of adolescent psychopathology is influenced by a number of psychological, environmental, and biological factors [1, 2]. According to the Developmental Origins of Health and Disease (DOHaD) hypothesis, signals from the mothers endocrine and immune system prepare the fetal organism for the after-birth-environment and thus modulate its development [3]. Accordingly, there are recent summaries reporting the association of prenatal stress with developmental impairment [4–7].

The prenatal environment can be influenced by a large variety of environmental and biological factors (see [8] for an overview). For example, prenatal stress, psychiatric symptoms and substance use by the mother can alter the endocrine levels [9], cortisol levels [10, 11], immune system functioning [12], microbiotic gut bacteria [13], or epigenetic expressions [14] in the intrauterine environment. While the association between adolescent psychiatric disorders and prenatal stress has been shown repeatedly [15–17], there is little research investigating the potential mechanisms. One candidate for a trans-diagnostic factor associated with detrimental prenatal influences is the capacity for self-regulation [18–20]. Based on the strong influence of prenatal factors on self-regulation capabilities, it is reasonable to assume that prenatal factors may be strongly associated with substance use disorders (SUDs) as well as cognitive functioning, both of which are intimately related to self-regulation capacities [21, 22]. However, it is important to note, that prenatal markers are only one aspect in multiple risk models for the developmental of psychiatric disorders, especially SUDs [23]. The older a child grows the more diverse and more complex psychological, biological and environmental factors are involved.

From a methodological standpoint, it is also crucial to consider how prenatal markers are assessed. One method is to collect data during pregnancy in the form of maternal self-reports or biomarkers. Importantly, objective (bio)markers have higher predictive value and are better predictors of child development than subjective reports [24, 25]. Another method involves retrospective study designs with objective data retrieved from medical records containing variables such as gestational age, birth weight, or Apgar scores as perinatal markers of prenatal health. However, these markers are highly unspecific compared to biomarkers that represent more defined processes and result in more specific variables, such as ethanol levels in the meconium [26] or facial abnormalities [27]. Nonetheless, unspecific general measures (gestational age, etc.) are easily obtained from medical records and have been shown to coincide with prenatal risks like maternal stress, depression, smoking, alcohol consumption or pregnancy illness [28–30]. Further, a disturbance in a number of retrospectively assessed perinatal markers is associated with an increased risk for child and adolescent psychopathology [31, 32], e.g. psychosis [33] and reduced cognitive functioning [34, 35]. Specifically, general intelligence is linked to self-regulation [21] and adolescent SUD is particularly marked by cognitive dysfunction [36]. Therefore, general intelligence might be associated with perinatal markers in SUD patients specifically.

In line with this literature and based on the idea that SUDs might be more strongly associated with prenatal development than other psychiatric disorders not as strongly related to self-regulation, we conducted the current study. The aim of this project was to explore if general perinatal markers can distinguish adolescent patients with a SUD from adolescent patients with other psychiatric disorders. Additionally, we explored the association between perinatal markers and continuous measures of psychopathology and a marker of cognitive functioning.

## Methods

### Procedure

Participants were recruited from two centers, one a specialized outpatient unit for adolescents with SUD (SUD sample) and one a general outpatient unit for children and adolescents (GEN sample). *SUD sample*: Data collection was embedded into standard diagnostic procedures. During the first clinical appointment, participants as well as legal guardians were asked to provide written informed consent for participation in this study. Psychopathological questionnaires were handed out during this first appointment. Cognitive testing was conducted approx. 1–4 weeks later. The study was conducted in accordance with the Declaration of Helsinki and all procedures were approved by the Institutional Review Board of the University Hospital C. G. Carus Dresden (EK 66,022,018). *GEN sample*: Data collection was performed retrospectively by study assistants retrieving individual data and intelligence test results from patients’ clinical records. Clinical records contain the data collected at time of admission at the general outpatient unit. In both samples, ICD-10 diagnoses were obtained by an assessment through experienced child and adolescent psychiatrists or psychologists.

### Participants

In the SUD center *n* = 196 participants agreed for their data to be included in the study, while *n* = 307 general center participants were added by retrieving their data from medical records. Table 1 displays the demographic details and prevalence of different disorders across the two centers.

**Table 1:** Demographic details of the two samples

	SUD group (*n* = 196)	GEN group (*n* = 307)	Test-statistic	p-value
Females (%)	76 (38.8)	163 (53.1)	*X²* (1) = 9.84	0.002*
Mean age	15.8 (1.4)	12.7 (3.5)	*t* (501) = 11.84	< 0.001*
N with ICD-10 Disorders^1^ (%)				
*F00-09*	1 (0.5)	0	*X²* (1) = 1.57	0.210
*F10-19*	163 (83.2)	7 (2.3)	*X²* (1) = 349.77	< 0.001*
*F20-29*	1 (0.5)	1 (0.3)	*X²* (1) = 0.10	0.749
*F30-39*	38 (19.4)	116 (37.8)	*X²* (1) = 19.06	< 0.001*
*F40-49*	37 (18.9)	152 (49.5)	*X²* (1) = 47.86	< 0.001*
*F50-59*	2 (1.0)	34 (11.1)	*X²* (1) = 18.20	< 0.001*
*F60-69*	10 (5.1)	9 (2.9)	*X²* (1) = 1.55	0.213
*F70-79*	2 (1.0)	2 (0.7)	*X²* (1) = 0.21	0.650
*F80-89*	3 (1.5)	17 (5.5)	*X²* (1) = 5.03	0.025*
*F90*	31 (15.8)	69 (22.5)	*X²* (1) = 3.33	0.068
*F91*	69 (35.2)	40 (13.0)	*X²* (1) = 34.65	< 0.001*
*F92*	3 (1.5)	23 (7.5)	*X²* (1) = 8.67	0.003*
*F93*	7 (3.6)	60 (19.5)	*X²* (1) = 24.43	< 0.001*
*F94*	1 (0.5)	9 (2.9)	*X²* (1) = 3.60	0.058
*F95*	0 (0)	16 (5.2)	*X²* (1) = 10.55	0.001*
*F98*	12 (6.1)	27 (8.8(	*X²* (1) = 1.19	0.274

*Note*: *significant at the 0.05 level; ^1^The corresponding disorders to the ICD-10 codes can be found in Table S1

As displayed in Table 1 above, the SUD sample shows a high prevalence of other psychiatric disorders, in many cases several psychiatric disorders at once. Since we aim to explore the difference in perinatal markers between a SUD sample and a general disorder sample, it is necessary to control for these coexisting disorders in the SUD sample. For this goal, we performed a manual 1-on-1 matching procedure, in which we matched participants in the SUD group with a participant from the GEN group according to age at study inclusion, gender, and specific pattern of coexisting disorders. Specifically, we searched for participants from each group with the same pattern of comorbidity (ICD-10 F10-19 disorders excluded) and the same gender. If several participants were available from a group, a match closest in age was selected. This resulted in a total sample of *n* = 102 participants (*n* = 51 from each center) since this strong matching procedure was only possible for few individual participants. Table 2 displays the demographic and diagnostic details of this matched sample.

**Table 2:** Demographic details of the matched sample

	SUD center (*n* = 51)	General center (*n* = 51)	Test-statistic	p-value
Females (%)	35 (68.6)	35 (68.6)	*X²* (1) = 0.00	1.000
Mean age	15.5 (1.6)	15.1 (1.8)	*t* (100) = 1.06	0.290
N with ICD-10 Disorders^1^ (%)				
*F00_09*	0 (0)	0 (0)	*X²* (1) = 0.00	1.000
*F10_19*	40 (78.4)	0 (0)	*X²* (1) = 65.81	<0.001*
*F20_29*	0 (0)	0 (0)	*X²* (1) = 0.00	1.000
*F30_39*	24 (47.1)	24 (47.1)	*X²* (1) = 0.00	1.000
*F40_49*	28 (54.9)	28 (54.9)	*X²* (1) = 0.00	1.000
*F50_59*	1 (2.0)	1 (2.0)	*X²* (1) = 0.00	1.000
*F60_69*	4 (7.8)	4 (7.8)	*X²* (1) = 0.00	1.000
*F70_79*	0 (0)	0 (0)	*X²* (1) = 0.00	1.000
*F80_89*	1 (2.0)	1 (2.0)	*X²* (1) = 0.00	1.000
*F90*	2 (3.9)	3 (5.9)	*X²* (1) = 0.21	0.647
*F91*	4 (7.8)	4 (7.8)	*X²* (1) = 0.00	1.000
*F92*	3 (5.9)	3 (5.9)	*X²* (1) = 0.00	1.000
*F93*	2 (3.9)	1 (2.0)	*X²* (1) = 0.34	0.558
*F94*	0 (0)	0 (0)	*X²* (1) = 0.00	1.000
*F95*	0 (0)	0 (0)	*X²* (1) = 0.00	1.000
*F98*	0 (0)	0 (0)	*X²* (1) = 0.00	1.000

*Note*: *significant at the 0.05 level; ^1^The corresponding disorders to the ICD-10 codes can be found in Table S1

### Materials

*Perinatal data*. In Germany, a number of perinatal measurements are recorded at birth and given to parents in the form of an individual report (“U-Heft”). Parents of SUD patients brought this report as part of the intake assessment. In the GEN sample, “U-Heft” data were retrieved retrospectively from routine medical records. From this report, we analyzed weeks of completed pregnancy including days of the final week (gestational age ‘GA’), birth weight in gram (‘weight’), birth mode (spontaneous vs. caesarean section (C-section)) and Apgar score after 5 min (APGAR5) [37].

*Cognitive functioning*. In both samples participants received a comprehensive assessment of general intelligence via the Wechsler Intelligence Scale for Children, fifth edition (WISC-V) [38]. This instrument provides users with a full-scale IQ as well as primary index scores, and subtest scores. The dependent variable (DV) from this measurement was the full-scale IQ.

*Psychopathology*. In the SUD sample, three measures of psychopathology were applied. In the Youth Self-Report (YSR/11–18) [39] adolescents rate their behavioral, emotional, social and physical problems in the previous six months on 120 items with three response options (not applicable = 0, sometimes = 1, frequently = 2). Answers can be summed up to a total higher-order scale resulting in a total behavioral problems score [40]. This score was the DV for our analysis. The German 16-Item short version of the Prodromal Questionnaire (PQ16) [41, 42] is a self-report questionnaire assessing the presence of attenuated psychotic symptoms on a two-point scale (true/false). The total score, our DV in this case, was calculated by the sum of positively answered symptoms (“true”) ranging from 0 to 16. The Drug Use Disorders Identification Test (DUDIT) [43], is a self-report instrument composed of 11 items identifying problems related to the use of illegal drugs, previously established for use in adolescents with SUD [44]. Items 1 to 9 of the DUDIT are scored on a five-point Likert scale, while items 10 and 11 are scored on a three-point scale (with the three items being scored 0, 2, and 4, respectively). The overall score is a sum of all items with a maximum score of 44. DUDIT total score was used as a DV.

### Statistical analysis

For our main analysis, we calculated a binary logistic regression with group (SUD vs. GEN) as a dichotomous outcome and GA, weight, APGAR5 and birth mode (spontaneous vs. C-section) as predictors. As a secondary analysis, we performed a linear regression in the matched sample to determine if the perinatal variables can predict IQ score. Additionally, we performed linear regression analyses in the original SUD and GEN samples to assess the influence of perinatal predictors on IQ (in the GEN sample) and IQ, DUDIT score, PQ16 score, YSR total score (in the SUD sample). The level of significance was defined as *p* < .05. To correct for multiple testing, significant p-values were adapted according to the Bonferroni-procedure.

## Results

### Perinatal differences between the SUD and GEN group

The binary logistic regression model predicting group membership (SUD vs. GEN) from GA, weight, APGAR5 and birth mode was not statistically significant (*X*^*2*^ [4] = 4.77, *p* = .312, Cox & Snell R² = 0.053). Neither did any single predictor reach statistical significance, see Table 3. Interpreting odds ratios indicates an effect of birth mode, with participants born through C-section being 2.46 times more likely to belong to the GEN group.

**Table 3:** Coefficients for the binary logistic regression predicting group (SUD vs. GEN) from perinatal markers

Measure	Unstandardized coefficient (SE)	Wald chi-square test	*p*-value	Bonferroni p-value	Odds ratio
Median weeks of pregnancy	0.229 (0.151)	*X*^*2*^ (1) = 2.29	0.130	n/a	1.26
Median birthweight in grams	− 0.001 (0.001)	*X*^*2*^ (1) = 3.34	0.068	n/a	1.00
Median Apgar score at 5 min	0.040 (0.344)	*X*^*2*^ (1) = 0.01	0.908	n/a	1.04
Birth mode	0.900 (0.665)	*X*^*2*^ (1) = 1.83	0.176	n/a	2.46

### Cognition, psychopathology and perinatal factors

The linear regression predicting IQ with perinatal variables was not significant in the matched sample (F [4] = 0.695, *p* = .600), the SUD sample (F [4] = 1.072, *p* = .376), or the GEN sample (F [4] = 2.058, *p* = .088). However, in the GEN sample, there was a statistical trend, with APGAR5 as the most relevant predictor (higher Apgar score, higher IQ score). Coefficients for each perinatal variable are displayed in Table 4.

**Table 4:** Coefficients for the analysis of IQ scores in total sample and each subsample

	Standardized coefficient	Unstandardized coefficient (SE)	Test statistic	p-value	Bonferroni p-value
IQ matched sample (*n* = 43), R² = 0.068					
*Birth mode*	0.059	1.925 (5.2)	t = 0.369	0.714	n/a
*Gestational age*	0.139	0.690 (1.3)	t = 0.518	0.608	n/a
*Birth weight*	0.083	0.002 (0.005)	t = 0.345	0.732	n/a
*APGAR5*	0.077	1.470 (3.7)	t = 0.402	0.690	n/a
IQ SUD sample (*n* = 82), R² = 0.053					
*Birth mode*	0.160	6.283 (4.4)	t = 1.435	0.155	n/a
*Gestational age*	0.096	0.298 (0.4)	t = 0.841	0.403	n/a
*Birth weight*	0.127	0.003 (0.003)	t = 1.119	0.267	n/a
*APGAR5*	0.011	0.189 (2.0)	t = 0.094	0.925	n/a
IQ GEN sample (*n* = 179), R² = 0.045					
*Birth mode*	0.019	0.460 (1.8)	t = 0.254	0.800	n/a
*Gestational age*	0.075	0.605 (0.8)	t = 0.731	0.466	n/a
*Birth weight*	-0.044	-0.001 (0.003)	t = -0.044	0.659	n/a
*APGAR5*	0.193	2.858 (1.1)	t = 0.193	0.013	0.052

*Note*: n/a = not available since the p-value was not significant at the < 0.05 level

In the SUD sample, the linear regressions between perinatal data and YSR (F [4] = 2.105, *p* = .091), DUDIT (F [4] = 0.617, *p* = .651), and PQ16 (F [4] = 0.508, *p* = .730) revealed no associations, see Table 5.

**Table 5:** Coefficients for psychopathological values in the SUD subsample

	Standardized coefficient	Unstandardized coefficient (SE)	Test statistic	p-value	Bonferroni p-value
YSR total score (*n* = 66), R² = 0.121					
*Birth mode*	-0.019	-0.800 (5.3)	t = -0.151	0.880	n/a
*Gestational age*	0.216	1.828 (1.2)	t = 1.594	0.116	n/a
*Birth weight*	-0.265	-0.007 (0.004)	t = -1.888	0.064	n/a
*APGAR5*	0.151	3.017 (2.5)	t = 1.197	0.236	n/a
DUDIT (*n* = 125), R² = 0.020					
*Birth mode*	-0.069	-1.967 (2.8)	t = -0.715-	0.476	n/a
*Gestational age*	0.088	0.263 (0.3)	t = 0.916	0.362	n/a
*Birth weight*	-0.099	-0.002 (0.002)	t = -1.029	0.306	n/a
*APGAR5*	0.039	0.487 (1.2)	t = 0.394	0.694	n/a
PQ16 (*n* = 100), R² = 0.021					
*Birth mode*	-0.116	-1.184 (1.1)	t = -1.087	0.597	n/a
*Gestational age*	0.055	0.053 (0.101)	t = 0.530	0.280	n/a
*Birth weight*	-0.031	0.000 (0.001)	t = -0.292	0.771	n/a
*APGAR5*	0.041	0.208 (0.527)	t = 0.395	0.694	n/a

*Note*: n/a = not available since the p-value was not significant at the < 0.05 level

## Discussion

In this cross-sectional study, we aimed to investigate the association between perinatal markers and adolescent psychopathology. We found that perinatal markers were not able to distinguish adolescent SUD patients from adolescent patients with other psychiatric disorders. Additionally, perinatal markers were not associated with full scale IQ in either the SUD or the GEN sample and were not associated with measures of SUD severity, attenuated psychotic symptoms, or general psychopathology. Interpreting effect size measures, data showed that C-section was more strongly associated with other disorders than SUD. While C-sections can be traumatic events for the mothers giving birth [45] the procedure is not associated with increased psychopathology [46]. However, children born by C-section show lower levels of externalizing symptoms [46] which can explain the lack of association between C-section and SUD, a disorder marked by externalizing behavior [47].

In addition, our results indicate that perinatal markers might not be associated with a self-regulation related disorder, specifically SUD, beyond the association with other psychiatric disorders. Similarly, no association was detected between the level of cognitive functioning and perinatal markers. On the one hand, these findings imply that the prenatal environment and adolescent health outcomes are not intimately related. On the other hand, our non-finding might be a reflection of the non-specificity of the analyzed markers and the focus on ICD-10 diagnoses instead of more fine-grained symptom constellations. More specifically, the included perinatal markers in our study are summary markers that can be influenced by a large number of prenatal factors [48, 49] and a single value might reflect the combined influence of various specific mechanisms. This means that our findings do not exclude the possibility of more specific markers, such as 2D:4D ratios [25, 50] or markers in the meconium [51], being associated with self-regulation related disorders.

An additional explanation is also available that might clarify our results and specifically the contrast to previous findings (e.g. [34, 35, 52, 53]). Generally, studies that report an association of psychopathology or cognitive ability with perinatal variables mostly consist of participants with extreme values on these perinatal variables. For example, Geller et al. [52] and Orri et al. [53] showed that obsessive-compulsive and affective disorders are associated with maternal illness and perinatal adversities requiring special care. Similarly, Uemura et al. [35] and Kroll et al. [34] showed reduced cognitive ability in samples of extremely low birth weight and preterm birth. However, the median values of the perinatal variables in our sample were comparable to normal data from the World Health Organization [54, 55] (e.g. WHO median birth weight = 3300 g compared to 3290 g in our sample, as well as WHO median gestational age = 39.43 weeks compared to 40.00 weeks in our sample). Therefore, the additional explanation of our unusual finding might be the fact that our sample showed very few outliers in terms of the perinatal variables (e.g. only 2.1% of participants had an Apgar score of below 7), which indicates that perinatal markers may only influence the development of psychopathology if they strongly deviate from the norm. Additionally, the comparison to data from the WHO shows that our sample of adolescents with SUD or other psychopathologies does not necessarily show signs of a disturbed prenatal environment. This is in line with the idea that psychiatric disorders are not the result of a single determinant but represent the result of a multiple vulnerability stress model. Since a child will encounter more potential environmental stressors the older it grows, these very early perinatal markers may be more meaningful in younger cohorts than in adolescents. Indeed, in a study with toddlers of 0–3 years of age perinatal markers showed a strong association with psychopathology [56]. Prenatal influences, as represented by perinatal markers, seem to be a single moving part in the complex interaction of biological, environmental and psychological factors that contribute to the development of adolescent psychopathology.

### Limitations and future research

First, while we specifically referred to SUDs in our manuscript our SUD sample was not exclusively afflicted by SUDs. Nearly all of our SUD participants fulfilled the criteria for at least one coexisting psychiatric disorder. This co-occurrence confounds the association between perinatal markers and SUDs we aimed to explore. However, by controlling for this co-occurrence through a strict matching procedure we were able to more accurately assess the association with SUDs specifically. Alternatively, a whole-sample regression analyses could have been applied with co-occurring disorders as predictors, which would have increased the sample size but also would have greatly increased the number of predictors. Therefore, we believe a 1-on-1 matching procedure to be more valuable in assessing the relationship between perinatal markers and SUDs.

Second, our data regarding the perinatal markers was obtained retrospectively. These variables were recorded by nurses working in a labor ward under pressuring circumstances, which might contribute to inaccuracy of recorded data. Additionally, the recording of these variables is not standardized procedure developed by the researchers but a record of clinical interest. Nonetheless, as mentioned in the introduction, objective data like this is still preferable to subjective assessments of prenatal disturbances [24, 25]. Future researchers might aim to obtain data during pregnancy and collect data themselves instead of analyzing retrospective medical records.

Third, self-regulation capabilities may not be as strictly linked to SUDs as we assumed. Indeed, there is evidence that self-regulation is related to a wide variety of child psychopathology [57]. However, even the GEN population did not show abnormalities in the perinatal markers compared to international standards [55], supporting our conclusion that we need more specific markers to investigate psychopathology in adolescence.

Fourth, our chosen perinatal markers are non-specific and disturbances in these markers can result as a large number of potential processes. An important result of this project is the affirmation of the need to obtain and analyze specific biomarkers that are related to specific mechanistic influences.

Fifth, we analyzed perinatal markers in a sample with high levels of psychopathology. If the perinatal markers are associated with psychopathology in general, distinguishing two affected groups become statistically more difficult and requires large samples. A future study would be well served by adding general population cohorts and by establishing differences in perinatal markers between pathologically affected and non-affected groups.

Further, a more fruitful research avenue could be longitudinal cohort studies. With this study design participants with high-risk perinatal markers could be followed up throughout their life to assess the risk of developing SUDs or other psychopathologies. Additionally, as mentioned above, our study investigated differences in diagnostic groups, meaning we examined the association between perinatal markers and ICD-10-based diagnostic groups. This focus, while clinically relevant, imposes the danger of losing detailed information on the symptom level. A more fitting analysis might be focusing on the association between perinatal markers and specific, neurobiologically defined constructs from the Research-Domain Criteria (RDoC) approach [58, 59]. Alternatively, more in line with our current study, a future project could investigate the associations between perinatal markers and adolescent mental health on the symptom level as defined by the Hierarchical Taxonomy of Psychopathology (HiTOP) system [60, 61] or based on network models of psychopathology [62, 63].

## Conclusion

Based on our results, we conclude that general perinatal markers might not be sufficient to investigate the association between the prenatal development and self-regulation-related disorders or general psychopathology. This study serves as a call to focus investigative efforts on specific, biological, and standardized markers of prenatal functioning. Additionally, associated psychopathology should be assessed and evaluated on the symptom level, based on network or hierarchical models that will lead to important trans-diagnostic conclusions regarding the development of child and adolescent psychopathology.

## Electronic supplementary material

Below is the link to the electronic supplementary material.

Supplementary Material 1. Psychiatric disorders and corresponding ICD-10 codes.

## Data Availability

The datasets used and analyzed during the current study are available from the corresponding author on reasonable request.

## Abbreviations

SUD: Substance use disorder.
HiTOP: Hierarchical Taxonomy of Psychopathology.
WHO: World Health Organization.
ICD-10: International Classification of Disease 10th Edition.
C-section: Caesarean section.
