# Supplementary material for: Are perinatal measures associated with adolescent mental health? A retrospective exploration with original data from psychiatric cohorts
Source: BMC Psychiatry. 2022 Oct 28;22:668. doi: 10.1186/s12888-022-04302-6 (PMC9617431; doi:10.1186/s12888-022-04302-6)
Supplement: Supplementary file 1 — Supplementary Material 1. Psychiatric disorders and corresponding ICD-10 codes. [file 12888_2022_4302_MOESM1_ESM.docx]

Are Perinatal Measures Associated with Adolescent Mental Health? A Retrospective Exploration with Original Data from Psychiatric Cohorts.

**Authors:** Lukas A. Basedow ^1,2^*; Sören Kuitunen-Paul ^1,3^; Veit Roessner^1^; Gunther H. Moll^4^; Yulia Golub ^1†^; Anna Eichler^4†^

* Corresponding author

† These authors contributed equally to the manuscript

^1^ Technische Universität Dresden, Faculty of Medicine, Department of Child and Adolescent Psychiatry, Fetscherstrasse 74, 01307 Dresden, Germany

^2^ Philipps-University of Marburg, Dept. of Psychology, Division of Clinical Psychology and Psychotherapy, Marburg, Germany

^3^ Technische Universität Chemnitz, Chair for Clinical Psychology and Psychotherapy, Chemnitz, Germany

^4^ Department of Child and Adolescent Mental Health, Faculty of Medicine, Friedrich-Alexander-Universität Erlangen-Nürnberg (FAU), Schwabachanlage 6, 91054 Erlangen, Germany.

**Table S1.** Psychiatric disorders and corresponding ICD-10 codes.

| F00-09 | Organic, including symptomatic, mental disorders |
| --- | --- |
| F10-19 | Mental and behavioral disorders due to psychoactive substance use |
| F20-29 | Schizophrenia, schizotypal and delusional disorders |
| F30-39 | Mood [affective] disorders |
| F40-49 | Neurotic, stress-related and somatoform disorders |
| F50-59 | Behavioral syndromes associated with physiological disturbances and physical factors |
| F60-69 | Disorders of adult personality and behavior |
| F70-79 | Mental retardation |
| F80-89 | Disorders of psychological development |
| F90 | Hyperkinetic disorders |
| F91 | Conduct disorders |
| F92 | Mixed disorders of conduct and emotions |
| F93 | Emotional disorders with onset specific to childhood |
| F94 | Disorders of social functioning with onset specific to childhood and adolescence |
| F95 | Tic disorders |
| F98 | Other behavioral and emotional disorders with onset usually occurring in childhood and adolescence |
